# Medical and Legal Risks in Tibial Plateau Fractures

**DOI:** 10.5811/cpcem.38452

**Published:** 2025-07-14

**Authors:** Rachel Lindor, Summer Ghaith, Jennifer Newberry, Aaron Thomas

**Affiliations:** * Mayo Clinic, Department of Emergency Medicine, Rochester, MN; † Mayo Clinic Alix School of Medicine, Mayo Clinic Phoenix, AZ; ‡ Stanford Health Care, Department of Emergency Medicine, Palo Alto, CA; § Mayo Clinic, Department of Emergency Medicine, Phoenix, AZ

**Keywords:** tibial plateau fracture, malpractice, lawsuit

## Abstract

**Introduction:**

Tibial plateau fractures, which comprise about 1% of all fractures, can be challenging to diagnose in the emergency department setting. Missed and delayed diagnoses can result in poor outcomes for patients and legal risks for clinicians, necessitating a high level of vigilance.

**Case Series:**

In this article we review three malpractice cases related to tibial plateau fractures. Key issues included missed or delayed diagnosis, mismanagement of associated complications, inadequate discharge instructions, and lack of documentation.

**Conclusion:**

Tibial plateau fractures can be challenging to identify, heightening the risk of downstream complications. As a result, emergency physicians must remain vigilant in assessing patients who are at increased risk for these injuries and document their efforts to both evaluate for and communicate these risks to patients.

## INTRODUCTION

Tibial plateau fractures account for 1% of all fractures, including 8% of fractures in patients ≥60 years of age.^1^,^2^ In younger populations, the most common mechanisms include motor vehicle collisions, sporting injuries, and high energy falls, while in the older population the most common mechanism is a ground level fall.^3^,^4^ Ultimately these forces result in articular depression and malalignment.^5^ Plain radiographs fail to identify approximately 20% of tibial plateau fractures, with complications of missed diagnoses or mismanagement ranging from chronic pain and disability to acute compartment syndrome and amputation.^6^

The low sensitivity associated with plain radiographs and the potential poor outcomes associated with tibial plateau fractures combine to make this a high-risk injury in the emergency department (ED) setting from both a medical and legal perspective.^7^ However, increased availability of computed tomography (CT) has improved detection when clinical suspicion remains high. Here, we examine three malpractice cases involving tibial plateau fractures, highlighting the key factors considered during litigation.

## CASE SERIES

### Case 1: *Sullivan*, California

A 27-year-old police officer presented to the ED after a high-speed pursuit of a suspect ended in a motor vehicle accident. Initial radiographs of his knee did not definitively identify a fracture, although it did show a fat-fluid level within the joint. Neither the treating emergency physician (EP) nor the consulting orthopedist ordered further testing. The patient was diagnosed with a knee sprain and instructed to bear weight as tolerated. Weeks later, due to persistent pain, he underwent repeat radiographs that revealed a significant fracture of the tibial plateau. The patient sued both the EP and the orthopedist for failing to diagnose his fracture on the radiographs and for allowing him to bear weight on the injury for so long, resulting in permanent pain and disability. This case was settled for $59,998 in 1986 (~$170,000 adjusted for inflation).^8^

### Case 2: *Reager*, West Virginia

A 13-year-old boy presented to the hospital with severe knee pain after falling from an 18-foot cliff and was found to have a tibial plateau fracture. Shortly afterward, his nurse reported to his physician that he had worsening lower leg pain, numbness, and a cold foot, but the physician did not return to re-evaluate him. Instead, he reportedly encountered an orthopedist at the elevator, verbally asked him to see the patient but did not document this request. The orthopedist did not recall the conversation, and the patient was not evaluated by either physician for the remainder of the night. By the time the patient was seen the next day, he was noted to have necrosis of a significant amount of his leg from a vascular injury and compartment syndrome, necessitating an amputation several days later. The patient alleged that the defendant physicians failed to diagnose this known complication, while the defendants contended that this was exceptionally unusual and that no intervention would have changed the outcome. This case went to trial and resulted in a verdict for the patient of $1,270,000 in 1984 (~$3.65 million adjusted for inflation).^9^

### Case 3: *Colchado*, California

A 35-year-old male presented to the ED after a fall and was diagnosed with a tibial plateau fracture by a radiograph. A long-leg splint was applied, and the patient was given morphine, diazepam, and ketorolac for pain. The defendant EP initially recommended that the patient be transferred to an in-network county hospital for evaluation of his knee. However, the patient refused transfer preferring instead to transfer to a private hospital and was ultimately discharged home with a referral for a next-day orthopedics follow-up appointment. The patient did not attend that appointment and four days later returned to the ED with worsening pain, where he was found to have compartment syndrome in his lower leg, necessitating multiple surgeries and resulting in permanent disability. The patient alleged that signs and symptoms of compartment syndrome were present at the time of his initial evaluation, that he should have been admitted for observation given this is a well-established complication, and that orthopedics should have been consulted.^10^ The initial trial resulted in a hung jury, and a second trial—more than four years after the incident—resulted in a verdict in favor of the physician, largely due to the patient’s refusal of transfer and failure to return.

## DISCUSSION

### Tibial Plateau Fractures: Clinical Pearls

Tibial plateau fractures can be difficult to diagnose in the ED setting and require a careful and well documented clinical approach. First, because they tend to occur in the setting of high energy trauma, the component of knee pain may be overlooked as secondary to other injuries. However, there are multiple exam findings that should increase suspicion of intra-articular pathology, such as tibial plateau fracture.^5^ Joint effusion, inability to fully extend the leg, or ecchymosis with obvious deformity when compared to the unaffected side are possible findings.^5^ Other indicators include inability to bear weight, difficulty raising the straight leg against gravity, and limitation of flexion-extension mechanism.^5^

*CPC-EM Capsule*

What do we already know about this clinical entity?

*Up to 20% of tibial plateau fractures are missed on initial imaging, creating the risk of serious long-term outcomes like chronic pain, compartment syndrome, or even amputation*.

What makes this presentation of disease reportable?

*These cases demonstrate how inadequate diagnosis, management, and documentation strategies have been tied to malpractice risks in previous cases of tibial plateau fractures*.

What is the major learning point?

*Tibial plateau fractures are easy to miss in the emergency department setting, requiring a high degree of clinical suspicion; understanding the clinical presentation, documenting an appropriate exam, and providing adequate follow-up care are essential to reducing the risk of bad outcomes*.

How might this improve emergency medicine practice?

*Improved recognition of tibial plateau fracture challenges can enhance patient care and potentially reduce clinicians’ liability risks*.

Second, because these fractures are often caused by torsion or impaction of the knee joint and present without classic overt signs of trauma, physicians often forego radiographs of the knee. In one study, researchers found that over half of patients with a tibial plateau fracture did not receive a knee radiograph in the ED.^1^ Use of a clinical decision rule among these patients would have significantly increased the frequency of plain radiography and identification of fractures.^1^ The Pittsburgh Knee Rule, used in this study, recommends radiographs following blunt trauma or fall in patients <12 or >50 years of age or in patients unable to walk four steps while weight-bearing. While the Pittsburgh Knee Rule has been demonstrated to have a sensitivity of 99% and specificity of 60% for identifying patients with fractures^11^,^12^ and these types of rules have been touted as a way to decrease unnecessary radiography, in the case of tibial plateau fractures, use of a decision rule may prevent physicians from missing these occasionally subtle fracture presentations.

An additional clinical challenge posed by tibial plateau fractures is the difficulty in visualizing these injuries on standard radiographs. Sensitivity of standard radiographs for these fractures is generally estimated to be around 80%, increasing to 85% with the addition of oblique views.^13^ However, in at least one study, fractures were missed in almost 40% of patients, possibly due to difficulty positioning patients appropriately for imaging.^1^ Many patients have subtle signs of fracture on radiographs, including non-alignment of the femoral condyles, presence of a joint effusion, or a fat/fluid level (lipohemarthrosis).^1^,^15^ Ultimately, in cases where radiographs do not reveal a tibial plateau fracture but clinical suspicion remains high, a CT or magnetic resonance imaging (MRI) should be considered as the next step in the diagnostic evaluation. Over the past several decades, MRI has emerged as the preferred imaging modality due to its ability to better characterize the fracture patterns and associated soft-tissue injuries, which aid in surgical planning. However, CT remains a viable option when MRI is unavailable, with both considered definitive tests for identifying significant fractures.^14^

Finally, disposition of patients with tibial plateau fractures poses risks, given the potential for downstream complications. In addition to associated compartment syndrome, these patients are at high risk of surgical complications as a result of associated meniscal and ligamentous injuries.^15^

### Tibial Plateau Fractures: Documentation Pearls

#### I. Discharge Instructions

Cases 1 and 3 highlight the importance of documenting clear discharge instructions. This is true for patients in whom tibial plateau fractures are confirmed, or even suspected, if definitive imaging is not available. Physicians are expected to communicate clearly with patients about the results of their testing, what changes would warrant a return visit or another evaluation, and how to manage their symptoms in the interim. In the first case, the patient was not told that it was a possibility that he had a knee fracture, and he was allowed to walk on his leg with the belief that it would improve with time, contributing to the development of his chronic symptoms. Failure of his treating physicians to recognize the poor sensitivity of radiographs for diagnosing tibial plateau fractures, to limit his weight-bearing, and to communicate and document this possibility of an occult fracture, likely contributed to the decision to settle this case out of court, as they could not argue that they met the standard of care.

In contrast, in the third case the patient was diagnosed with a tibial plateau fracture but had a delayed presentation of compartment syndrome, also resulting in chronic symptoms; however, the physicians in his case had arranged for him to have follow-up the next day, had documented this plan, and were able to show that they had met the standard of care regarding return precautions; ultimately it was determined to be the patient who failed to follow the recommendations.

Although physicians often emphasize documenting the details of clinical encounters, effective communication with patients during and after discharge is also crucial for reducing liability risks. Legal actions in this area may arise from unclear referrals, inadequate discharge instructions, insufficient return precautions, or a lack of follow-up on pending test results.^16^ Investing time in discussing *and documenting* post-discharge care recommendations may help reduce physicians’ exposure to these types of lawsuits.^17^. In patients with diagnosed or suspected tibial plateau fractures, discharge instructions should include non-weight bearing with the use of assistive devices such as crutches, walkers, or wheelchairs. Splinting or supportive braces may be prescribed for comfort. Close orthopedic follow-up is of utmost importance. Lastly, discussion of the signs of compartment syndrome must be discussed, among them skin color changes, loss of sensation, increase in pain, and loss of distal pulses.

#### II. Discussions with Consultants

Case 2 highlights the importance of appropriately documenting formal consultations. The EP in this case contended that he abided by the standard of care by consulting an orthopedist to assist in the patient’s care when he was alerted to developing compartment syndrome, but he did not document this consult and there was no record that this ever occurred. Malpractice cases for EPs related to consultations can be mitigated by adhering to a well-defined protocol for formal consultations, including documenting the consultant’s name, the time and relevant details of the discussion, and ensuring that consultants understand that their recommendations will be used for patient care.^16^ Of course, in this case, the treating physician’s failure to return to the bedside highlighted his inattention to the patient and prevented him from recognizing that the consult did not happen. Taking continued responsibility for patients regardless of how many people have been consulted on their behalf is also key to reducing liability exposure.

## CONCLUSION

Our review of three malpractice cases associated with tibial plateau fractures reveals insights into the challenges of timely and accurate diagnosis and the medico-legal risks associated with this diagnosis. Better recognizing the clinical challenges associated with tibial plateau fracture diagnosis and management can help clinicians improve patient care, communicate more clearly with patients and consultants, and mitigate their liability risks.
